# Associations Between Health Information Sources, Sociodemographic and Clinical Characteristics, and Decision-Making Among Women with Early-Stage Breast Cancer

**DOI:** 10.21203/rs.3.rs-9657916/v1

**Published:** 2026-06-17

**Authors:** Caroline Salafia, Keith M. Bellizzi, Timothy E. Moore, Elizabeth A. Hintz, Jolaade Kalinowski, Patricia A. Parker

**Affiliations:** Memorial Sloan Kettering Cancer Center; University of Connecticut; University of Connecticut; University of Connecticut; University of Connecticut; Memorial Sloan Kettering Cancer Center

**Keywords:** women with breast cancer, early-stage breast cancer, surgical decision-making, health information, information sources, information seeking

## Abstract

**Background:**

Most breast cancer cases in the United States are diagnosed early, and women often must decide whether to undergo mastectomy or breast-conserving surgery to treat their cancer. Ideally, women engage in shared decision-making with their provider, which involves becoming informed about surgical options. This study examined where women obtain health information to inform surgical decision-making, sociodemographic and clinical factors associated with their use of specific sources, and potential factors (e.g., cancer stage) associated with surgical decisions.

**Methods:**

Women diagnosed with early-stage breast cancer (*N* = 194) completed an online survey assessing the health information sources they used to inform their surgical treatment decision.

**Results:**

Women relied on various sources, including healthcare providers, the internet, social media, and friends and family. The most frequently cited were breast cancer surgeons, breast cancer organizations, Facebook, and spouses/partners. Women of color had higher odds (*OR* = 2.99, 95% CI = [.03, 2.16]) of utilizing information from friends and family, whereas women with more advanced disease had lower odds (*OR* = .59, 95% CI = [.09, .96]). Women who were married, had a family history of breast cancer, and used social media were more likely to have had a mastectomy.

**Conclusions:**

These findings highlight the multifaceted nature of information-seeking and decision-making among women with early-stage breast cancer and underscore the need for continued research as the health information landscape evolves.

## Background

Approximately 1 in 8 women in the United States (U.S.) will be diagnosed with breast cancer during their lifetime [1]. When breast cancer is detected at an early stage (localized to the breast and/or axillary lymph nodes), the 5-year relative survival rate exceeds 98% [2]. Approximately 66% of breast cancer cases in the US are diagnosed at an early stage [3]. Consequently, most women diagnosed with early-stage breast cancer can expect long-term cancer survival, joining the more than 4 million survivors of breast cancer living in the U.S. [4].

For women with early-stage breast cancer, surgery is the standard of care [5]. Both breast-conserving surgery (BCS) and mastectomy are well-established and effective surgical treatment options [5]. BCS, also referred to as lumpectomy, involves the removal of the tumor and surrounding breast tissue and is typically followed by radiation therapy [6]. In contrast, mastectomy involves the removal of the entire breast to treat the cancer [7]. Extensive research has demonstrated no significant differences in survival or recurrence rates between BCS followed by radiation therapy and mastectomy for early-stage breast cancer [8–11]. Therefore, surgical treatment for early-stage breast cancer is considered “preference-sensitive,” as multiple clinically appropriate options exist and decisions should incorporate patients’ individual goals, values, and preferences [12, 13]. Accordingly, many women engage in shared decision-making with their healthcare providers to weigh the risks and benefits of each surgical option and play an active role in determining their course of treatment [14].

### Shared Decision-Making and Preference-Sensitive Medical Decisions

Over the past few decades, the healthcare system has shifted from a more paternalistic style of medicine (the doctor tells the patient what to do) toward a collaborative decision-making approach [15, 16]. This process is known as shared decision-making, an approach in which healthcare providers and patients share the best available evidence when making medical decisions, and patients are encouraged to consider their options and make informed choices about their care [17]. Shared decision-making is a fundamental communication challenge and is particularly relevant to the surgical management of breast cancer, as women are presented with two surgical treatment options that result in overall equivalent prognoses in terms of survival and rate of local recurrence [18–20]. Deciding on the best course of action can be challenging, given that patients have diverse needs and preferences regarding their treatment. Still, shared decision-making is an encouraged care delivery model, particularly in the context of preference-sensitive medical decision-making, as it is associated with increased satisfaction with care, informed decision-making, and less decisional regret following cancer treatment [21–25]. Preference-sensitive decisions, such as the surgical treatment decision for early-stage breast cancer, require patients to weigh the advantages and disadvantages of each surgical approach. Healthcare providers often focus on treatment efficacy and side effects, while patients consider a broader set of quality-of-life factors [26]. Each surgical option has differing benefits, risks, and impacts on women’s short and long-term quality of life [27, 28]. For instance, one benefit of BCS is that more of the breast is preserved, but one benefit of mastectomy is that women may be able to avoid radiation therapy treatment after surgical treatment [28]. In order for shared decision-making to take place in the context of the surgical treatment for breast cancer, individuals must be informed about their treatment options [18]. One of the ways women diagnosed with breast cancer become informed about their surgical treatment options is by utilizing health information from a variety of sources.

Preference-sensitive decisions require access to sufficient information to support informed choice [13]. To aid in their decision-making, many women seek out health information to understand their cancer and treatment options [29, 30]. The rapid expansion of digital media has greatly increased access to health information, enabling women to become more informed and actively participate in surgical decision-making [31, 32]. Women with early-stage breast cancer draw on a variety of information sources, including healthcare providers, the internet, social media platforms (e.g., Facebook), other women with breast cancer, and friends and family [33–37]. Historically, healthcare providers and the internet have been the most frequently cited sources, although emerging evidence suggests an increase in the use of breast cancer–specific social media communities, such as Reddit [34, 37, 38]. Given the rapidly evolving digital information landscape, updated research is needed to identify which information sources women most commonly use when making surgical decisions.

### Factors in Health Information Source Utilization and Surgical Decision-Making

The use of health information in breast cancer surgical decision-making varies across personal and contextual factors, including sociodemographic, clinical, and personality characteristics. More specifically, access to and use of online health information is influenced by age, race, ethnicity, education, and health literacy [39]. Younger, more educated individuals are more likely to engage with online health information [40], while individuals with low health literacy are less likely to seek cancer-related health information [41]. Racial and ethnic differences also exist, with non-Hispanic White individuals more likely to use the internet for health information compared to Black and Hispanic individuals [42]. Lastly, individual differences in personality influence information-seeking behavior, with openness associated with greater engagement in online health information seeking [43, 44].

In addition to health information, sociodemographic and clinical factors can influence surgical treatment decisions. Previous research has found that having a family history of breast cancer and having already breastfed a child are associated with a preference for mastectomy [45], and less invasive breast cancer is a predictor of choosing BCS [46]. Some research has not found an association between age and receiving a mastectomy [47], while other studies suggest that younger women are more likely to receive a mastectomy [48, 49]. Given the mixed findings, future research is needed to explore factors associated with surgical treatment decisions. Additionally, because surgical treatment for early-stage breast cancer is a preference-sensitive decision for many women, it may be important to assess how the use of specific sources of information (e.g., the internet) may be associated with one’s surgical treatment decision. Given the prognostic equivalence of both surgeries, BCS is often the recommended treatment of early-stage breast cancer by physicians, given that it is less invasive [50–52]. However, many women in the United States still opt for mastectomy despite being a candidate for BCS [53, 54]. While many factors influence a woman’s surgical treatment decision, it is also imperative to assess how specific sources of health information may influence their decision. Recent research has found that family members are influential in women’s decisions to undergo a mastectomy [55]. Understanding the potential relationship between health information sources and surgical treatment decisions may provide healthcare providers with insights into how women make preference-sensitive decisions about their cancer care.

Accordingly, the present study sought to build on existing knowledge by further examining health information-seeking and surgical decision-making among women diagnosed with early-stage breast cancer. Specifically, this study aimed to address three research questions (RQs):

#### RQ1

What sources of health information do women with early-stage breast cancer use to inform their surgical treatment decisions?

#### RQ2

Which sociodemographic, clinical, and personality characteristics are associated with the utilization of specific health information sources?

#### RQ3

What factors (i.e., source utilization, sociodemographic, and clinical characteristics) are associated with women’s surgical treatment decisions?

Identifying the information sources women rely on, the factors associated with source utilization (e.g., stage of breast cancer, personality), and the determinants of surgical treatment choice may enhance understanding of how women navigate preference-sensitive medical decisions following an early-stage breast cancer diagnosis and inform efforts to support shared, patient-centered decision-making.

## Methods

### Participants and Procedures

**Figure 1:**
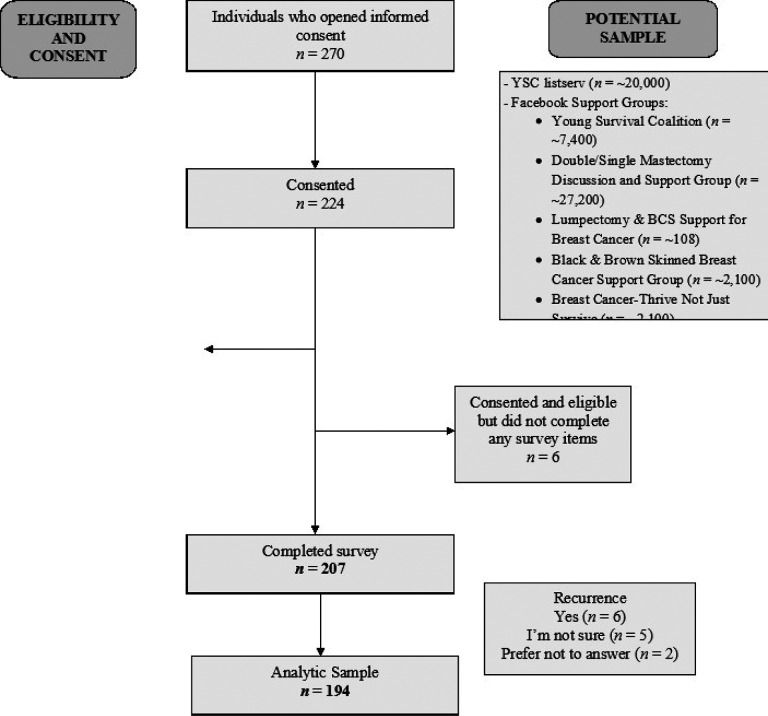
Flowchart describing the recruitment for the study

This study was part of an observational, retrospective investigation examining surgical decision-making among women diagnosed with early-stage breast cancer. Participants were recruited in 2024 through breast cancer-related support groups on Facebook, with administrator approval (see Fig. 1), and through the Young Survival Coalition (YSC), a nonprofit organization serving young women diagnosed with breast cancer. To reduce fraudulent responses, a CAPTCHA bot-detection screener was administered prior to eligibility screening in Qualtrics.

Women were eligible to participate if they: 1) resided in the U.S.; 2) were 18 years of age or older; 3) had been diagnosed with early-stage breast cancer; 4) were candidates for either BCS or mastectomy; and 5) had undergone surgical treatment (i.e., BCS vs. Mastectomy) between one and five years prior to study participation. Eligible participants completed a 10–15-minute online survey administered via Qualtrics. Participants who completed the survey were invited to enter a raffle for one of five $50 Amazon gift cards. All study procedures were approved by the Institutional Review Board at the University of Connecticut.

## Measures

### Demographic Characteristics

Sociodemographic characteristics were collected via self-report, including age at breast cancer diagnosis and age at time of study participation. Participants also reported their educational attainment, marital status, and employment status. Race and ethnicity were assessed based on guidance from federal data collection and reporting [56]; participants selected their race from a predefined list (e.g., select all that apply, Black, White, Asian, American Indian or Alaska Native, Other) and indicated ethnicity as either non-Hispanic or Hispanic.

### Clinical Characteristics

Participants self-reported the stage of breast cancer at diagnosis and the number of years since surgery. They also indicated whether they had undergone genetic testing prior to surgery. For those who reported prior genetic testing, participants were asked whether they tested positive for a breast cancer-related gene mutation (e.g., *BRCA1/2, ATM, CHEK2, PALB2*)?” with response options of “Yes,” “No,” “I’m not sure,” or “Prefer not to answer.” Family history of breast cancer was assessed using a “select all that apply” format listing first- and second-degree relatives (e.g., mother, grandmother, sister, aunt, etc.), along with an option for no family history. Participants further reported whether they had received radiation, chemotherapy, immunotherapy (e.g., Keytruda), hormone therapy (e.g., Tamoxifen), or other treatments [57, 58].

#### Personality Characteristics

Personality was assessed using the 10-item Big Five Inventory-10 (BFI-10 [59]), which evaluates five personality traits: extraversion, agreeableness, conscientiousness, neuroticism, and openness to experience. Participants rated items on a five-point scale from 1 (*disagree strongly*) to 5 (*agree strongly*) in response to prompts beginning with “I see myself as someone who…” Items were reverse-scored as appropriate, and trait scores were calculated by summing the relevant items, with higher scores indicating greater levels of each trait.

#### Surgical Treatment Decision

Participants reported the type of surgical treatment they received using categories defined by the American Cancer Society [60]. They were provided with brief descriptions of BCS and mastectomy and selected the option corresponding to their initial breast cancer surgery. Participants who selected “other” could specify their surgical procedure in a text box.

### Health Literacy

Health literacy was measured using the 3-item Brief Health Literacy Screen (BHLS [61]). Items assess participants’ difficulties understanding written health information and confidence in comprehending medical information, rated on 5-point Likert scales. Item responses were summed to yield total scores ranging from 3 to 15, with higher scores indicating greater subjective health literacy. Internal consistency in the current sample was good (α = .82).

### Health Information Source Utilization

Informed by prior research [62–64], participants reported whether they used information from four broad categories: 1) healthcare providers, 2) the internet, 3) social media, and 4) friends and family. Examples were provided for each category (e.g., oncologist for healthcare providers, breast cancer organization websites for the internet, Facebook for social media, and spouse for friends and family). Participants who indicated use of a category were then presented with a detailed “select all that apply” list of specific sources (see Table 2), including additional healthcare providers (e.g., genetic counselors) and social media platforms (e.g., Facebook, Reddit). Participants could also select “other” and specify additional sources not listed.

### Data Management and Analysis

Data management was conducted continuously from recruitment through study completion. Data were downloaded from Qualtrics at 10-participant intervals to identify potential issues (e.g., bots, spam) and ensure quality. Data were exported to SPSS (Version 30) for cleaning. Missing data were minimal, ranging from 0.5% to 1.0% across primary study variables, and analyses were conducted using listwise deletion. Participants with a recurrence of breast cancer were excluded, as their health information-seeking behaviors and surgical treatment experiences may differ.

Descriptive statistics were used to examine the frequency of utilization of health information sources. Binary logistic regression analyses were conducted to evaluate associations between sociodemographic and clinical variables and the binary response: the use of each information source (yes/no, with non-use as the reference). Additional logistic regressions assessed the associations between sociodemographic and clinical characteristics, information source utilization, and surgical treatment decisions (mastectomy vs. BCS, with BCS as the reference). Statistical significance was set at *p* < .05. All statistical analyses were run in SPSS Version 30.

## Results

Table 1 presents the descriptive statistics of the analytic sample, which consisted of 194 women diagnosed with breast cancer. The mean age at diagnosis was 43.65 years (*SD* = 9.15) and 45.62 (*SD* = 9.16) at study completion. Most participants identified as White (76.8%) and non-Hispanic (96.2%). The majority were diagnosed with stage 1 breast cancer (41.2% of participants), followed by stage 2 (27.8% of participants), and most participants had undergone surgery within the last two years (*M* = 1.79, *SD* = 1.57).

Nearly all participants reported receiving genetic testing (93.3%), but most (85.9%) did not test positive for a breast cancer-related gene. At the time of study participation, 64.4% had undergone breast reconstruction. A total of 36% of participants received radiation in the past, 40% received chemotherapy in the past, and 10% received immunotherapy in the past. Just over half were currently receiving hormone therapy (53.9%). Self-reported health literacy was high in the sample (*M* = 13.62, *SD* = 1.91, range 5–15).

### Health Information Source Utilization

Addressing RQ1, participants reported using multiple sources of health information to inform their surgical treatment decisions. The most commonly utilized sources were healthcare providers (96.9%), followed by the internet (79.7%), friends and family (65.6%), and social media (63.0%). Among healthcare providers, breast cancer surgeons were consulted most frequently (93.8%), followed by oncologists (60.8%), and genetic counselors (29.9%).

The most frequently reported internet sources were breast cancer foundations and organizations (63.4%), government websites (46.9%), and hospital or cancer center websites (41.2%). Among family and friends, spouses or partners were most commonly cited (44.3%), followed by friends with cancer (34.0%) and mothers (32.0%). With respect to social media, Facebook was the most frequently used platform for health information (58.2%), followed by Instagram (12.4%) and YouTube (8.8%). A complete list of health information sources is presented in Table 2.

### Individual Factors Associated with Health Information Source Utilization

Addressing RQ2, binary logistic regression analyses were conducted to examine associations between sociodemographic, clinical, and psychosocial factors and the likelihood of using specific health information sources (internet, social media, and friends and family) vs. non-use. Predictors included cancer stage, age at diagnosis, education, family history of breast cancer, race, positive genetic test results, marital status, years since surgery, health literacy, and personality characteristics.

The overall models for internet (*p* = .48) and social media use (*p* = .65) were not statistically significant, suggesting that the combined set of predictors did not significantly improve model fit relative to the intercept-only model, and no individual sociodemographic, clinical, or personality variables were associated with utilization of these sources (all *p* > .05). The overall model predicting utilization of friends and family was not statistically significant (*p* = .11). However, within the model, two predictors showed statistically significant association with utilization. Given the non-significant omnibus test, these individual effects should be interpreted cautiously. Higher cancer stage was associated with lower odds of using friends and family as an information source (*b* = − .52, *SE* = .22, *p* = .02; *OR* = 0.59). In addition, race was significantly associated with friends and family utilization, such that women of color had nearly three times higher odds of using friends and family for health information compared to White women (*b* = 1.10, *SE* = .54, *p* = .04; *OR* = 2.99). All other predictors, including family history, marital status, positive genetic test results, age at diagnosis, years since surgery, education, health literacy, and personality characteristics, were non-significant (all *p* > .05; see Table 3).

### Predictors of Surgical Treatment Decision

Finally, addressing RQ3, a logistic regression model was used to examine factors (i.e., source utilization, sociodemographic, and clinical characteristics) associated with the likelihood of receiving a mastectomy versus breast-conserving surgery. The overall model was statistically significant (*χ*^2^(9) = 17.85, *p* = .04) and demonstrated modest explanatory power, with a Nagelkerke pseudo-R^2^ of .15.

Family history of breast cancer, marital status, and the utilization of social media for health information emerged as significant predictors. Women with a family history of breast cancer had more than twice the odds of undergoing a mastectomy compared to those without a family history (*OR* = 2.48, *b* = .39, *p* = .02). Similarly, married women had higher odds of receiving a mastectomy than unmarried women (*OR* = 2.69, *b* = .47, *p* = .03). Use of social media as a health information source was also associated with increased odds of mastectomy, with users nearly three times as likely to undergo mastectomy compared to non-users (*OR* = 2.84, *b* = .44, *p* = .02). Positive genetic test results, age at diagnosis, race, and utilization of other health information sources were not significantly associated with surgical treatment decision (all *p* > .05; see Table 4).

## Discussion

The present study sought to enhance understanding of health information seeking and preference-sensitive surgical decision-making among women diagnosed with early-stage breast cancer. Specifically, the study examined the health information sources women used to inform their surgical treatment decisions, identified sociodemographic, clinical, and personality characteristics associated with each information source, and evaluated factors associated with surgical treatment choice. Collectively, these findings contribute to a more nuanced understanding of how women navigate complex, preference-sensitive surgical decisions in the context of an evolving health information landscape.

### Health Information Sources Used in Surgical Decision-Making

The findings indicate that women with early-stage breast cancer rely on a broad and overlapping set of health information sources when making surgical treatment decisions, with most participants reporting use of all four categories examined (healthcare providers, the internet, social media, and friends and family). Nearly all participants reported obtaining information from healthcare providers, underscoring the central role healthcare providers—particularly breast cancer surgeons and oncologists—continue to play in preference-sensitive decision-making. Importantly, participants also sought information from other providers, including genetic counselors, nurses, and primary care providers, highlighting the multidisciplinary nature of surgical decision-making and the opportunity for consistent, coordinated communication across care teams.

Most participants (80%) reported using the internet to inform their decisions, suggesting increased reliance on digital information compared to earlier estimates, which found that 30–40% of individuals with cancer sought health information online [65–67]. The most commonly used internet sources were breast cancer foundations and government websites, which are generally considered credible and evidence-based, and may reflect the high educational attainment of the sample. These findings underscore the importance of ensuring that reputable organizations continue to provide accessible, patient-centered information to support informed decision-making.

Social media also emerged as a commonly used information source, particularly platforms such as Facebook and Instagram, reinforcing the growing role of peer-to-peer information exchange in cancer care. Among friends and family, the most frequently reported source was a spouse/partner (44%), consistent with prior research demonstrating the influential role of intimate partners in cancer-related decision-making [68]. Additionally, many women sought information from friends who had experienced breast cancer themselves, aligning with evidence that patients value experiential knowledge and shared understanding from others with similar diagnoses [69]. Together, these findings highlight the increasingly complex information environment in which surgical decisions are made and point to the need for healthcare providers to acknowledge, discuss, and help contextualize information patients obtain from multiple sources.

Given that the surgical treatment for early-stage breast cancer is often a preference-sensitive decision that ideally encourages shared decision-making, it is critical that women are informed about the surgical treatment options and the advantages and disadvantages of each surgery. The findings from the present study indicate that women seek a variety of sources (i.e., the internet, social media, friends, and family) to inform their surgical treatment decision. These findings highlight the need for effective communication between women and healthcare providers to address unmet information needs, additional questions and concerns, and potential exposure to misinformation that may be influencing surgical treatment decision-making. A recent study found that individuals diagnosed with cancer who were exposed to a patient-centered approach to communication during oncology appointments reported greater trust in healthcare providers, along with stronger intentions to both seek out and discuss online health information with providers, compared to those exposed to a provider-centered communication approach [70]. Shared decision-making and patient-centered care can be improved by encouraging women to share which information sources they’ve utilized and how these sources have informed their values and preferences for treatment. By integrating health information-seeking behavior into clinical encounters and patient-provider communication, more effective, informed, and preference-concordant surgical decision-making may occur.

### Sociodemographic and Clinical Differences in Information Source Utilization

The present study found that women of color had almost three times higher odds of using friends and family as sources of health information to inform surgical treatment decisions compared to White women. This finding is consistent with prior research demonstrating racial and ethnic differences in information-seeking and decision-making preferences. For example, a cross-sectional study of 200 women diagnosed with breast cancer found that Black and Latina women rated information from healthcare providers, such as medical records and pathology reports, as less helpful for treatment decision-making than White women [71] and that the family played a particularly influential role among Latina women. Similarly, Wallner and colleagues [72] found that White women were more likely than women of color to use the internet and social media for breast cancer-related health information.

These patterns may reflect broader structural and relational factors, including medical mistrust, which has been identified as a significant barrier to relying on healthcare providers for health information, particularly among Black women in the United States [73]. Supporting this interpretation, data from the Health Information National Trends (HINTS) survey indicate that, among the general population, Black individuals are less likely than White individuals to use health information from healthcare providers [74]. Although nearly all participants (97%) in the present study reported using information from healthcare providers, these findings may differ in more racially and ethnically diverse samples. Future research with larger and more diverse populations is needed to better understand how race, trust, and cultural norms shape information-seeking and surgical decision-making.

In addition to race, cancer stage was associated with use of friends and family as information sources, such that the likelihood of relying on these sources decreased as disease stage increased. This finding is not surprising and highlights that individuals may be less likely to seek out information from their social networks if their cancer is more advanced and may prioritize information from healthcare providers. Together, these findings underscore the importance of culturally responsive, trust-building communication strategies and highlight the need to tailor decision-support resources to patients’ informational preferences and clinical contexts.

### Information Sources, Misinformation, and Surgical Treatment Choice

Surgical decision-making for early-stage breast cancer is inherently complex, as women are often presented with two clinically appropriate options—BCS and mastectomy—that differ in risks, benefits, and implications for quality of life [27, 28]. Understanding how women’s information-seeking behaviors influence these decisions is therefore critical, particularly in an increasingly digital information environment. Prior research suggests that many women feel inadequately informed about their surgical options, with more than half of women undergoing lumpectomy and one-third of those undergoing mastectomy reporting insufficient information prior to surgery [75].

In the present study, use of social media for health information was associated with significantly higher odds of receiving a mastectomy. Although causal inferences cannot be drawn from this cross-sectional design, this finding raises important questions about the nature and framing of information encountered on social media platforms. Recent research indicates that misinformation is widespread, with nearly 80% of women with breast cancer reporting exposure to inaccurate information [76]. Moreover, analyses of breast cancer–related discussions on Reddit suggest that sentiment toward mastectomy has become increasingly positive over time, whereas discussions of BCS have remained largely neutral [77].

Although the present study did not assess the content or sentiment of information sources, these findings suggest that certain platforms may differentially frame surgical options in ways that influence patient preferences. Future mixed-methods research is needed to examine not only where women seek health information, but also how information quality, framing, and emotional tone shape surgical decision-making.

Several limitations should be considered when interpreting the findings of this study. First, the cross-sectional, retrospective design limits causal inferences, including conclusions about the relationship between social media use and surgical treatment decisions. Participants were asked to recall their decision-making processes from approximately 1 to 2 years earlier, and their responses may be susceptible to recall bias. Additionally, reliance on self-reported data raises the possibility of social desirability bias, particularly if participants underreport use of less traditional information sources, such as social media, relative to healthcare providers, who are often viewed as more credible. Online recruitment through social media platforms also presents limitations, including potential sample bias, and limited representativeness (e.g., excluding women who do not use Facebook), privacy concerns, and technology-related barriers [78]. Finally, the generalizability of the findings is limited, as the sample was relatively small and predominantly composed of highly educated, non-Hispanic White individuals. Future research should explore the utilization of health information sources in a larger, more diverse sample of women affected by breast cancer.

## Conclusions

This study illuminates how women with early-stage breast cancer navigate a complex landscape of health information when making preference-sensitive surgical decisions. Most women drew on multiple sources, including healthcare providers, the internet, social media, and friends and family, highlighting the multifaceted nature of patient information-seeking. Emerging media forms, including social media platforms, online patient communities and forums, and artificial intelligence and chatbots (e.g., ChatGPT), are poised to play a growing role in shaping how patients access, interpret, and apply health information and may ultimately influence how women with early-stage breast cancer access, interpret, and use health information in their surgical decision-making. Future research should examine the influence of these new media on decision-making processes and outcomes.

Importantly, these findings underscore the central role of healthcare providers in guiding patients through diverse sources of information. By fostering open, supportive discussions, providers can help verify information, correct misconceptions, and address concerns, ultimately strengthening trust and promoting informed, patient-centered decision-making. Integrating these discussions into clinical care can enhance alignment between patients’ information needs and treatment decisions, improving overall surgical decision-making experience for women diagnosed with early-stage breast cancer.

## Figures and Tables

**Table 1:** Sample characteristics (*N* = 194)

Variables	*M* or *N* (SD, Range, or %)
Age at diagnosis	43.65 (9.15, 26–74)
Age at time of survey	45.62 (9.16, 26–74)
Stage of breast cancer	
Stage 0	46 (23.7%)
Stage 1	80 (41.2%)
Stage 2	54 (27.8%)
Stage 3a	11 (5.7%)
Another stage 3	3 (1.5%)
Race	
White	139 (76.8%)
Black or African American	26 (14.4%)
Asian	6 (3.3%)
Mixed Race	5 (2.8%)
Other	4 (2.2%)
Ethnicity	
Non-Hispanic	175 (96.2%)
Hispanic	7 (3.8%)
Marital Status	
Married/Partnered	143 (79.0%)
Not Partnered	38 (21%)
Education	
High school diploma/GED	8 (4.4%)
Some college/associate degree	42 (23.1%)
Bachelor's degree	53 (29.1%)
Graduate or Professional Degree	79 (43.4%)
Employment status	
Employed	149 (81.8%)
Unemployed or other	23 (12.6%)
Retired	9 (4.9%)

**Table 2:** Percent of participants that utilized specific health information sources to inform their surgical treatment decision

Source of Information	*n* (%)
**Healthcare Providers**	
Breast Cancer Surgeon	182 (93.8)
Oncologist	118 (60.8)
Genetic Counselor	58 (29.9)
Nurses	47 (24.2)
Primary Care Provider	47 (24.2)
Another Physician/Healthcare Provider	52 (26.8)
Patient Navigator/Healthcare Coordinator	43 (22.2)
Radiologist	38 (19.6)
Other	5 (2.6)
**General Internet Sources**	
Breast Cancer Foundation/Organization	123 (63.4)
Government Website	91 (46.9)
Hospital or Cancer Center Website	80 (41.2)
Online Scientific Journal Article	65 (33.5)
Other	27 (13.9)
News Website	20 (10.3)
**Social Media Sources**	
Facebook	113 (58.2)
Instagram	24 (12.4)
YouTube	17 (8.8)
Reddit	13 (6.7)
TikTok	12 (6.2)
Other	7 (3.6)
X (Twitter)	2 (1.0)
**Friends and Family**	
Spouse/Partner	86 (44.3)
A friend that had cancer	66 (34.0)
Mother	62 (32.0)
Friends	61 (31.4)
Sister(s)	38 (19.6)
A family member that had cancer	36 (18.6)
Extended family	29 (14.9)
Father	21 (10.8)
Coworkers	19 (9.8)
Member of a faith-based community	14 (7.2)
Daughter(s)	13 (6.7)
Brother(s)	9 (4.6)
Son(s)	5 (2.6)
Other	1 (0.5)

**Table 3:** Logistic regression of factors associated with using friends and family for health information

Variable	B	S.E.	OR	95% CI	*p*
Family history	−.30	.40	.74	−1.07, .48	.46
Marital status	.43	.50	1.54	−.51, 1.37	.37
Positive gene	.03	.58	1.03	−1.10, 1.15	.97
Race	1.10	.54	2.99	.03, 2.16	**.04**
Age	−.02	.02	.98	−.02, .07	.32
Stage of cancer	−.52	.22	.59	.09, .96	**.02**
Years since surgery	−.15	.13	.86	−.10, .39	.24
Health literacy	.12	.11	1.13	−.33, .08	.25
Intercept^a^	1.53	2.37	.04	−3.13, 6.19	.52

*Note*. B = unstandardized coefficient; 95% CI = 95% confidence intervals for the odds ratio.

a Reference group = Friends and family not utilized; Not married; No genetic mutation; White women. Education level (coded into four levels, with the lowest education level as the reference group) and all personality traits (extraversion, agreeableness, conscientiousness, neuroticism, and openness) were not significant (*p* > .05).

**Table 4:** Logistic regression of factors associated with choosing mastectomy vs BCS

Variable	B	S.E.	OR	95% CI	*p*
Family history	.91	.39	2.48	−1.68, −.14	**.02**
Marital status	.99	.47	2.69	−1.90, −.07	**.03**
Positive genetic test	.04	.58	1.04	−1.17, 1.10	.95
Race	−.40	.46	.67	−.50, 1.30	.38
Age	−.02	.02	.99	−.03, .06	.50
Stage of breast cancer	−.19	.22	.83	−.23. .62	.37
Internet	−.68	.53	.51	−.35, 1.67	.20
Social media	1.05	.44	2.84	−1.91, −.18	**.02**
Friends and family	.01	.41	1.01	−.78, .83	.99
Intercept^a^	.51	1.24	1.67	−3.91, .77	.68

*Note*. B = unstandardized coefficient; 95% CI = 95% confidence intervals for the odds ratio.

a Reference group = Breast-conserving surgery (BCS); No family history; Not married; No genetic mutation; White women; Health information sources not utilized.

## Data Availability

The dataset analyzed during the current study is available from the corresponding author on reasonable request.

## Abbreviations

BCS: Breast-conserving surgery
RQ: Research question
YSC: Young Survival Coalition
